# A-002 (Varespladib), a phospholipase A_2 _inhibitor, reduces atherosclerosis in guinea pigs

**DOI:** 10.1186/1471-2261-9-7

**Published:** 2009-02-17

**Authors:** Jose O Leite, Ushma Vaishnav, Michael Puglisi, Heather Fraser, Joaquim Trias, Maria Luz Fernandez

**Affiliations:** 1 Department of Nutritional Sciences, University of Connecticut, Storrs, CT, USA; 2 Anthera Pharmaceuticals, Inc. 25801 Industrial Blvd Hayward, California, USA

## Abstract

**Background:**

The association of elevated serum levels of secretory phospholipase A_2 _(sPLA_2_) in patients with cardiovascular disease and their presence in atherosclerotic lesions suggest the participation of sPLA_2 _enzymes in this disease. The presence of more advanced atherosclerotic lesions in mice that overexpress sPLA_2 _enzymes suggest their involvement in the atherosclerotic process. Therefore, the sPLA_2 _family of enzymes could provide reasonable targets for the prevention and treatment of atherosclerosis. Thus, A-002 (varespladib), an inhibitor of sPLA_2_enzymes, is proposed to modulate the development of atherosclerosis.

**Methods:**

Twenty-four guinea pigs were fed a high saturated fat, high cholesterol diet (0.25%) for twelve weeks. Animals were treated daily with A-002 (n = 12) or vehicle (10% aqueous acacia; n = 12) by oral gavage. After twelve weeks, animals were sacrificed and plasma, heart and aorta were collected. Plasma lipids were measured by enzymatic methods, lipoprotein particles size by nuclear magnetic resonance, aortic cytokines by a colorimetric method, and aortic sinus by histological analyses.

**Results:**

Plasma total cholesterol, HDL cholesterol and triglycerides were not different among groups. However, the levels of inflammatory cytokines interleukin (IL)-10, IL-12 and granulocyte-macrophage colony-stimulating factor (GM-CSF) were significantly reduced in the treatment group. This group also had a significant 27% reduction in cholesterol accumulation in aorta compared with placebo group. Morphological analysis of aortic sinus revealed that the group treated with A-002 reduced atherosclerotic lesions by 24%.

**Conclusion:**

The use of A-002 may have a beneficial effect in preventing diet-induced atherosclerosis in guinea pigs.

## Background

Atherosclerosis is a major component of cardiovascular disease [1]. There is evidence that inflammation not only aggravates the damages of hypercholesterolemia but also participates in the genesis of atherosclerosis [2-5]. Usually, in a steady state, there is a homeostatic balance between agonist and antagonist mediators of inflammation. However, when lipids and oxidized lipids deposit in the arterial wall, a protective mechanism follows, which disrupts the equilibrium and favors a cellular and humoral response. Inflammatory markers such as high-sensitivity C-reactive protein (hs-CRP), serum amyloid A, interleukin-6 (IL-6), and soluble intercellular adhesion molecule type 1 (sICAM-1), are predictors of risk in patients with cardiovascular disease [6].

Chronic inflammation associated with atherosclerosis can be monitored by the concentration of inflammatory cytokines, enzymes and other markers, among them secretory phospholipases A_2 _(sPLA_2_). Elevated levels of plasma type IIA sPLA_2 _or PLA_2 _activity are predictors of cardiovascular events [7-9]. For example, the increase in the concentration or activity of this enzyme is an independent risk factor for coronary artery disease and is associated with a three-fold increase in such risk [7,9,10] and suggest a role of sPLA_2 _in atherosclerosis [1,4].

Secretory PLA_2 _is a group of calcium-dependent enzymes that hydrolyze phospholipids at the *sn*-2 position producing free fatty acids and lysophospholipids [11]. This class of PLA_2 _enzymes share a unique catalytic dyad that allows for the design of inhibitors that will specifically inhibit sPLA_2 _enzymes but will have no effect on other PLA_2 _enzymes that do not have this catalytic dyad (for example cPLA_2_, iPLA_2_, or Lp-PLA_2 _enzymes) [12]. There are approximately 10 groups of sPLA_2 _enzymes that differ in their expression and distribution or substrate affinity. For example, in humans, type IB sPLA_2 _is secreted by acinar cells from pancreas and has its major function in digestion [13]. In contrast, type IIA sPLA_2 _is produced in spleen, thymus and other organs. This type of sPLA_2 _has been implicated in inflammatory response and in the development of atherosclerosis [13].

Evidence supporting the importance of sPLA_2 _enzymes in atherosclerosis include their co-localization in atherosclerotic lesions, their presence in proximity of lipid deposits in the arterial wall and genetic linkage between pro-atherogenic LDL particles and sPLA_2 _haplotypes (including IIA, IID, IIE, IIF, III, V, and X) [14-17]. A lower phosphatidylcholine (PC):lysophosphatidycholine (LPC) ratio (substrate and product of the sPLA_2 _enzymatic reaction respectively) in atherosclerotic areas also indicates that this family of enzymes is active in atherosclerotic lesions [1,18]. Experiments with transgenic mice that overexpress human type IIA sPLA_2 _demonstrated increased atherosclerosis compared to nontransgenic littermates [19]. Even more striking are the findings from Webb *et al *[15]. These authors showed that macrophage – expressed type IIA sPLA_2 _mice had an increase in atherosclerosis compared to the control group. Bostrom *et al *[20] demonstrated that type V sPLA_2 _can also contribute to the development of atherosclerosis. The researchers demonstrated that the transplantation bone marrow cells from either type V sPLA_2_^+/+ ^or type V sPLA_2_^-/- ^mice to LDL receptor deficient mice (LDL^-/-^) promoted different degree in atherosclerosis onset. Type V sPLA_2_^+/+ ^animals presented larger atherosclerotic lesions in aorta compared to control animals. In addition, type V sPLA_2_^-/- ^mice had a reduction of 36% in atherosclerotic lesion area compared to type V sPLA_2_^+/+ ^mice.

Recent findings have also implicated type X sPLA_2 _in atherosclerosis. Hanasaki et al. [21] demonstrate this enzyme is expressed in foam cells in apo-E deficient mice. The authors also showed that type X sPLA_2 _hydrolyzes phospholipids from LDL and releases more arachidonic acid much more efficiently than group IIA sPLA_2_. They also observed cholesterol accumulation in macrophages in the presence of type X sPLA_2_, suggesting that this enzyme induces the uptake of modified LDL.

Given the association between atherosclerosis and sPLA_2_, the inhibition of sPLA_2 _is a reasonable target for the discovery and development of novel cardiovascular drugs. Thus, the objective of this study is to evaluate the possible benefit of A-002, varespladib methyl (LY-333013 or S-3013), an oral prodrug of A-001 (LY-315920 or S-5920) an inhibitor of sPLA_2_, in preventing atherosclerosis in guinea pigs. A-001 is an indole derived compound discovered by rational drug design targeting the active site of sPLA_2_, and it is a broad and potent inhibitor of sPLA_2 _enzymes, including IIA, V, or X, with IC_50_s in the low nM range [22-25] with no effect on cytoplasmic PLA_2 _(cPLA_2_)[22]. Snyder *et al*[22] have demonstrated that A-001 efficiently inhibits sPLA_2 _activity in several species such as rat, rabbit and guinea pig. They also showed that the contractile response of the guinea pig lung pleural strip, which is mediated by TXA_2_, was suppressed by A-001. However, when it was challenged with arachidonic acid, A-001 was not effective. These findings suggest that the action of A-001 was upstream of arachidonic acid, blocking its liberation. Therefore, we speculate that A-002 will play a similar role. The inhibition of sPLA_2 _by A-002 will reduce the liberation of arachidonate. In consequence, it will reduce the production of eicosanoids by lipooxygenase and cyclooxygenases. A-002 may still mitigate the production of platelet-activating factor by inhibiting the release of lysophospholipids. Despite the fact that A-002 does not have any effect in the inhibition of cPLA_2_, an enzyme that provides arachidonic acid for eicosanoids synthesis, we expect that the inhibition of sPLA_2 _will interfere with the cross-talk between cPLA_2 _and sPLA_2 _and consequently, it will lead to a reduction in the production of arachidonate metabolites. We also speculate that the sPLA2 mass will be reduced such as suggested by Rosenson *et al*.[26] The authors demonstrated that the treatment with varespladib resulted in a greater reduction in type IIA sPLA_2 _mass compared to placebo group.

The guinea pig was the animal model chosen because they express sPLA_2 _[27] and also develop atherosclerosis when challenged with a high cholesterol, high saturated fat diet [27,28].

## Methods

### Animals

Twenty-four male guinea pigs, weighing from 280 to 300 g, were subjected to an *ad libitum *atherogenic diet (high saturated fat and high cholesterol diet) as previously described [29]. Guinea pigs will not develop atherosclerosis in the absence of this diet [28], which was required to test the effect on A-002 on atherosclerosis. The composition of the diet is indicated in Table 1. Guinea pigs were randomly assigned into 2 groups (12 animals per group) a control group that received vehicle and a treatment group that received A-002. Vehicle was 10% acacia (Sigma-Aldrich, St. Louis, MO) in water. A-002 was prepared to a final concentration of 30 mg/mL in vehicle.

**Table 1:** Composition of experimental diets for control (gavaged with saline) or treatment guinea pigs (gavaged with A-002).

	**g/100 g**	**% Energy**
**Soybean protein**	22.5	23

**Carbohydrate**	40.5	35

**Fat mix^a^**	15.1	42

**Cellulose**	10.0	

**Guar gum**	2.5	

**Mineral Mix^b^**	8.2	

**Vitamin Mix^b^**	1.1	

**Cholesterol**	0.25	

^a^The fat mix was high in lauric and myristic acids known to cause endogenous hypercholesterolemia in guinea pigs.

^b^Mineral and vitamin mix are formulated to meet NRC requirements for guinea pigs.

In both groups, animals were subjected to daily gavage for 3 months. The amount of drug per kilogram (150 mg/kg/day) was kept constant throughout the study. Animals were sacrificed in a CO_2 _chamber, blood was collected by heart puncture and the heart and aorta were harvested for analysis.

The dose of A-002 used in the present study was selected based on previous studies that showed adequate serum levels of A-001 for the duration of the study in guinea pigs [28].

### Plasma lipids and lipoprotein subfractions

Plasma lipids were measured by enzymatic methods [30-32] using standard kits from Roche-Diagnostics (Indianapolis, IN – USA). For total cholesterol, a Roche cholesterol (CHOD-PAP) assay that is standardized by CDC (Centers for Disease Control and Prevention – USA) was used. The first step of this experiment is based on the conversion of cholesteryl-ester (CE) to cholesterol by cholesterol-esterase. The following reaction is the oxidation of cholesterol, which generates hydrogen peroxide (H_2_O_2_). Finally, the reaction between a color reagent and H_2_O_2 _catalyzed by peroxidase produces a specific intensity of color that is proportional to the amount of cholesterol.

The plasma HDL-C assay is based on the analysis of the cholesterol that is left in the specimen after the precipitation of apo B-containing lipoproteins. Plasma triglycerides (TG) were measured using a triglycerides/glycerol blanked (Trig/GB) assay from Roche-Diagnostics (Indianapolis, IN – USA). LDL cholesterol (LDL-C) was calculated by the Friedewald equation as described by Flynn *et al*.[33].

Nuclear magnetic resonance (NMR) analysis was performed on a 400 MHz NMR analyzer (Bruker BioSpin Corp, Billerica, MA), as previously described[15] Briefly, lipoprotein subclasses of different sizes produce a distinct lipid methyl signal whose amplitude is directly proportional to lipoprotein particle concentration. Size ranges used for the LDL subfractions were: large LDL (21.2–23 nm), medium LDL (19.8–21.2 nm) and small LDL (18–19.8 nm).

### Cholesterol in aorta

Approximately 0.05 g of abdominal aorta was dissected and all surrounding tissues were removed. The vessel was kept overnight in 10 mL of chloroform:methanol (2:1) solution for lipid extraction. The solution was filtered in Whatman grade No. 1 filter paper and mixed with 3 mL of sulfuric acid at 0.05%. The lower phase was adjusted with chloroform:methanol (2:1) solution to a final volume of 10 mL. Thus, 200 μL was aliquoted, evaporated and resuspended in 200 μL of ethanol. This material was analyzed by enzymatic methods using standard kits from Roche-Diagnostics (Indianapolis, IN, USA).

### Cytokines concentration in the aorta

Cytokines were evaluated from homogenized descendent thoracic aorta, such as described by Sharman *et al *[34]. Briefly, the vessel was dissected and all the surrounding tissues were removed. The vessel was mixed and homogenized in a rotor-stator with 1 mL of lysis buffer (0.1 g of bovine serum albumin, 5 μL of triton-x 100, 100 mg of gentamycin sulfate, 100 μL of hepes buffer – 1 M, 23 μL of aprotinin, 18.391 mg of sodium ortovanadate and PBS to complete 1 mL). After this, 2 mL of the lysis buffer was added to the content and this was homogenized in a Potter-Elvehjem tissue grinder. This was centrifuged at 400 × g for 10 minutes at 4°C. The supernatant was analyzed by LINCOplex™ Cyokine Kit (Linco Research Inc, St. Charles, MO – USA) in a Luminex instrument (Luminex 200 System, Austin, TX.) according to the manufacturers' specifications.

### Arterial morphology

For the analyses of atherosclerosis, hearts and aortas were immersed in formalin, and paraffin sections at 3–5 μm were obtained from these tissues. Slides were stained with hematoxylin and eosin and they were evaluated via light microscopy by digital image analysis on aortic sections to assess plaque formation. Plaque formation in an aortic sinus was scored by a board certified pathologist based on a 6-point severity-scoring system (the greater the score, the more severe the atherosclerosis). The amount of plaque, open lumen area, and the total lumen area of the internal elastic lamina (IEL) were measured to calculate both plaque:lumen ratio and plaque:IEL ratio. Data was analyzed by the histology module of LABCAT software (version 8.0; Innovative Programming Associates, Inc., Princeton, NJ).

### Statistical analyses

Independent t-tests were used to compare the means of the parametric variables (plasma lipids, aorta cholesterol, cytokines concentration, plaque:lumen ratio and plaque:IEL ratio) between treatment and control groups. For the non-parametric variable, the atherosclerosis score, the Mann-Whitney test was used. Pearson's correlations were used to evaluate the correlations between LDL particle size and inflammatory cytokines. P < 0.05 was considered statistically significant.

## Results

**Figure 1:**
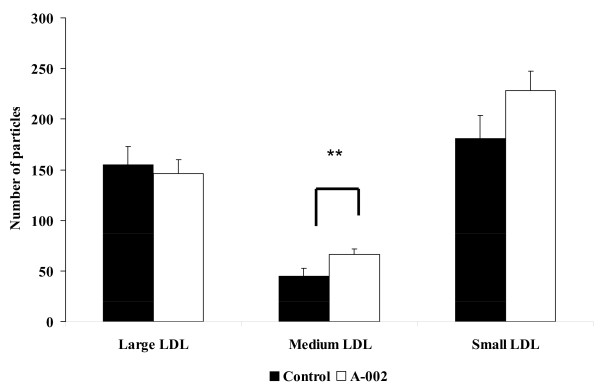
**Plasma concentrations of large, medium and small LDL in guinea pigs fed the control (dark bar) or the A-002 (white bar) diet**. Values are presented as mean ± SEM for 10 animals per group. ** indicates significantly different from control.

Plasma total cholesterol, LDL-C, triglycerides and HDL-C levels were not statistically different between A-002-treated and control groups (p > 0.05). Plasma lipid concentrations for both groups are presented in Table 2. The results from the LDL particles are described in Figure 1. Large and small LDL particle concentrations were not statistically different between treatments. However, medium LDL particle concentration was significantly lower in guinea pigs from the control group (p < 0.05).

**Table 2:** Plasma concentrations total, LDL and HDL cholesterol of guinea pigs treated with the drug or placebo (control group)^1^

**Plasma Lipids**	**Control**	**A-002**	**P Value**
Total Cholesterol (mg/dL)	237.2 ± 21.2	249.1 ± 14.9	0.65

Triglycerides (mg/dL)	48.5 ± 4.3	54.5 ± 4.9	0.372

HDL-Cholesterol (mg/dL)	17.6 ± 3.3	19.3 ± 2.5	0.696

LDL-Cholesterol (mg/dL)	209.8 ± 22.1	218.8 ± 17.1	0.749

^1 ^Values are expressed as mean ± SE for n = 10 guinea pigs per group.

**Figure 2:**
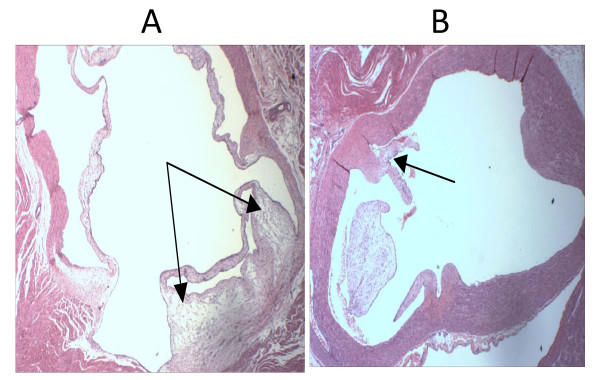
**Atherosclerotic plaque in aortic sinus sections**. Both sections are stained with hematoxylin-eosin, and presented at 40× magnification. A) Control group with mild to moderate plaque is present at the base of the valves. B) A-002 group with minimal plaque is present at the base of the valves. The dark arrows demonstrate the foam cells.

The amount of cholesterol expressed as mg/g in aorta was 25% lower in guinea pigs treated with A-002 than in the control group (Table 3). Although there was an important difference in the percentage of reduction (24% reduction in severity score measured by digital image analysis) in atherosclerosis from control compared with the treatment group, these differences did not reach statistical significance. Animals from the A-002-treated group exhibited a trend in reducing aortic sinus atherosclerosis compared with the control group. The results are presented in Figure 2.

**Table 3:** Concentration of cholesterol in aorta and morphological analyses of aortic sinus of guinea pigs treated with the drug or placebo (control group)^1^

**Parameter**	**Control**	**A-002**	**% Reduction from control**	**p-value**
Aorta Cholesterol (mg/g)	8.34 ± 0.78	6.05 ± 0.32	27%	0.025

Aortic sinus plaque formation (score)	2.1 ± 0.260	1.6 ± 0.318	24%	0.221

Plaque:lumen ratio (%)	4.27 ± 0.708	2.63 ± 0.317	38%	0.146

Plaque:IEL ratio (%)	4.04 ± 0.651	2.55 ± 0.302	37%	0.154

^1 ^Values are expressed as mean ± SEM for n = 10 guinea pigs per group.

**Figure 3:**
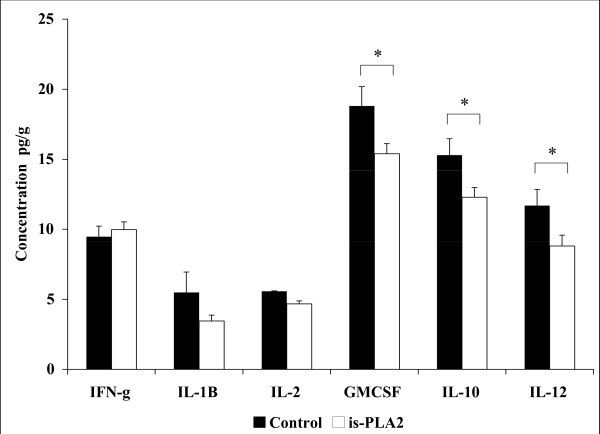
**Aortic concentrations of inflammatory cytokines in guinea pigs fed the control (dark bar) or the A-002 (white bar) diet**. Values are presented as mean ± SEM for 10 animals per group. * indicates significantly different from control.

**Figure 4:**
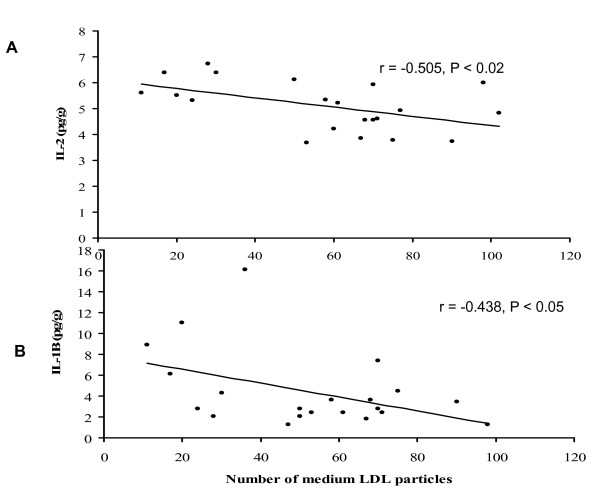
**Panel A: Correlation between the number of LDL particles and the plasma concentration of interleukin-2 (IL-2) for all guinea pigs**. Panel B. Correlation between the number of LDL particles and the plasma concentration of interleukin-1B (IL-1B).

The level of aortic cytokines was lower in the A-002-treated group. Levels of granulocyte-macrophage colony-stimulating factor (GM-CSF), interleukin-10 (IL-10) and interleukin-12 (IL-12) were significantly lower in the A-002-treated group. In contrast, interferon-gamma (IFN-g), interleukin-1B (IL-1B) and interleukin-2 (IL-2) levels were not statistically different between treated and non-treated groups (Figure 3). Although there was not a statistically significant difference between groups with respect to IL-1B and IL-2, both were negatively correlated with medium LDL particles (Figure 4A and 4B). In addition, the total numbers of particles was significantly negatively correlated with IL-12 (r = – 0.479; p = 0.018) and IL-2 (r = – 0.541; p = 0.011).

## Discussion

Treatment with A-002 (varespladib), an oral prodrug of the sPLA_2 _inhibitor A-001, did not change the plasma lipid profile of treated animals compared with the control group in this study. However, treatment with A-002 in mice fed high cholesterol diet has been associated with a significant decrease in total cholesterol levels [35] and human subjects treated with A-002 also show a decrease in LDL cholesterol [26].

Even though there was no reduction in plasma lipids in guinea pigs, the drug had significant effects on atherosclerosis, and on lipid levels in the aorta. The significant 27% reduction in cholesterol accumulation in aorta and the trend to reduce the formation of foam cells in the A-002 group (demonstrated in morphological analyses) support the idea that A-002 might help in the prevention of atherosclerosis, and longer treatment could have amplified the response. There is evidence that the lipid trapping of lipoproteins in arterial wall is modulated by the action of sPLA_2 _enzymes and its effect seems to be cumulative [36,37].

The mechanisms suggested to explain the reduction in the atherosclerotic processes are based on the inhibition of the action of sPLA_2_. Sartipy *et al*[38] demonstrated that sPLA_2 _type IIA is found in arterial wall bound to decorin proteoglycans, being co-localized to apo-B containing lipoproteins. The authors observed that the binding of sPLA_2 _type IIA to decorin increases the phospholipids hydrolyses of these lipoproteins more than two fold. A-002 reduced the breakdown of phospholipids and decreased the liberation of pro-inflammatory free fatty acids and LPC. The latter lipids promote alterations in the function of vascular cells that make them more susceptible to atherosclerosis, independently of changes in plasma lipids [3,4]. In addition, Hernandez et al.[39] have shown that secretory sPLA_2_stimulates inflammation. Beyond its catalytic effect, sPLA_2 _can also induce inflammation through cell membranes receptors. For example, the interaction of sPLA_2 _with muscle-type receptor may stimulate mitogen-activated protein kinase cascade, activates cPLA_2_, induces cyclooxygenase – 2 (COX-2) expression, promotes the monocyte differentiation into macrophage, among other effects[40,41] Whether A-002 is able to inhibit the sPLA_2 _binding to M receptor or not still needs further analyses. However, treatment with A-002 was able to reduce inflammatory cytokines. Inflammation promotes an increase in arterial wall permeability to atherogenic particles by the disruption of endothelium, thus, cholesterol is more prone to accumulate[42,43] In the present study, although we did not see a difference in small and large LDL particle concentration between groups, the number of medium LDL particles was significant higher in the A-002 group compared to control. In addition, medium LDL was negatively correlated with IL-1B and IL-2. These findings suggest that in the A-002 group, the effects of the drug can reduce the delipidation of the LDL by lipases, keeping the LDL in a larger particle size (medium size), which is less atherogenic[4,44] In addition, during inflammation, the endothelial barrier is more permeable, which can favor the accumulation of larger LDL particles [45,46]. This is consistent with the observation in the control group, which had higher levels of aortic inflammatory markers and higher lipid levels in the aorta. On the other hand, in the A-002 group, the lower inflammation status made the endothelial barrier less permeable to larger LDL particles. Several reports show that the increase in the retention of lipoproteins in extracellular matrix (ECM) facilitates their uptake by macrophages[4,36,47] This lipid retention associated with increased macrophage activity favors the appearance of foam cells [4,36,47]. Thus, in our study, the reduction in inflammation in the aorta in the A-002 group might help to prevent cholesterol accumulation in the arterial wall through the decrease in endothelial permeability. It is also possible that sPLA_2 _also reduced the levels of PC and LPC in the arterial lesion and decreased the expression of sPLA_2 _in the arterial wall as these components precede the production of inflammatory cytokines [48], which were reduced in this study. Further studies are necessary to confirm these findings.

In the present analyses, there was a statistically significant reduction in lipid accumulation in aorta between the control and the A-002 group. However, the morphological analyses of the atherosclerotic lesion showed just a decreasing trend compared to the experimental group. Most atherosclerotic lesions were classified as mild or moderately severe, even in the control group. This suggests that in the animal samples used, the atherosclerotic process was incipient. According to the literature, the lipid accumulation in the aorta precedes the appearance of macrophages and, consequently, of foam cells [14,37,49]. Thus, the difference between the lipid accumulation in the aorta wall and the morphological analyses in our study might have been a result of the incipient levels and low severity of atherosclerosis. In arterial walls, lipid accumulation is found not only intracellularly in foam cells, but also in ECM and type IIA sPLA_2 _may be involved [21,37]. Thus, while in morphological analyses only intracellular lipids were evaluated, in the Folch method, both intra and extracellular lipids were measured. The larger pool of lipids in the entire aortic tissue compared to that found in atherosclerotic lesions could explain the incongruence in the results of the two analyses [4,14,37,49].

In general, the reduction in inflammatory aortic cytokines indicates a lower degree of arterial wall inflammation. The inhibition of sPLA_2 _reduces the activation of cPLA_2 _and decreases the liberation of arachidonic acids and its products[50,51] Although we could not find statistical difference in IFN-g, IL-1B and IL-2 levels in the aorta, the concentrations of IL-10, IL-12 and GM-CSF were significantly lower in the A-002 treated group compared with control.

IL-12 is one of the cytokines that was significantly lower in the A-002 group. This cytokine has been involved in the early stages of atherosclerosis and with activation of T lymphocytes in response to atherogenic stimuli [52]. The reduction of IL-12 observed in the A-002 group represents a decrease in pro-atherosclerotic factors in the initial stage of this process.

GM-CSF was also significantly reduced in the A-002 group. This inflammatory cytokine is associated with cellular migration and proliferation in atherosclerotic process [53,54]. Recent studies have demonstrated the association between GM-CSF, and worsening of atherosclerotic lesions [53-55] For example, animals subjected to atherosclerotic diet exacerbate atherosclerosis development after administration of GM-CSF [55]. The authors demonstrate that the combination of high fat diet and high levels of GM-CSF synergistically increases atherosclerosis in murine models[53,55] Therefore, it might be possible that the reduction in GM-CSF levels by A-002 could also have contributed to prevent the development of atherosclerosis.

The IL-10 concentration in aorta was unexpectedly lower in the A-002 group. This interleukin is a prototype anti-inflammatory cytokine, and has been reported as a modulator of IL-12 action [56]. Typically, IL-10 inhibits the expression of IL-12. However, in our study, we found that both IL12 and IL-10 were decreased in A-002 group. IL-10 is produced in response to an inflammatory stimulus represented by the higher levels of IL-12. This can be supported by the chronological observation that IL-12 appears before IL-10 in atherosclerotic lesion [56]. A possible explanation for our data is that since in the control group the lipid accumulation in aorta was more severe, the inflammation was more pronounced. Thus, the level of IL-10, to try to modulate this inflammation, was also higher in the control group. On the other hand, in the A-002 group, IL-12 levels were reduced. Therefore, lower levels of IL-10 were required to modulate the inflammatory process in the treatment group.

An explanation for why the other cytokines did not significantly differ between groups, may be because the animals had incipient atherosclerosis, thus, it is possible that the degree of inflammation was not pronounced enough to be able to detect differences.

The limitations of the study were related to the severity of atherosclerosis. Maybe a longer study or the use of older animals may have amplified the efficacy signal with clearly established atherosclerosis. In addition, a pre-conditioning intervention before the drug treatment may help to produce greater levels of inflammation pre-intervention. This might make more evident the anti-inflammatory effect of the drug.

## Conclusion

The results suggest that the mechanisms by which A-002 might contribute to prevent the development of atherosclerosis are related to the reduction in lipid retention due to changes in LDL subfractions and not to decreased LDL cholesterol in this animal model. Another possible mechanism of action that helps in the prevention of atherosclerosis is the reduction of inflammation. Thus, A-002 may have a beneficial effect in preventing diet-induced atherosclerosis.

## Abbreviations

HDL-C: HDL cholesterol; IEL: internal elastic lamina; LDL-C: LDL cholesterol; LPC: lisophosphatidylcholine; NMR: nuclear magnetic resonance; PC: phosphatidylcholine; sPLA_2_: secretory phospholipase A_2_; TC: total cholesterol.

## Competing interests

Authors received funding from Anthera Pharmaceuticals, Inc. (San Mateo, CA) to carry out the studies presented in this manuscript.

## Authors' contributions

JOL did the assays, wrote the manuscript and participated in the interpretation of data; MJP and UV: assisted in the assays for plasma lipids, cytokine measurements and participated in data interpretation, HF and JT assisted in data interpretation, study design, review of the manuscript and data analysis and MLF designed the experiment, evaluated the results, interpreted the data and participated in manuscript preparation. All authors read and approved the final manuscript.

## Pre-publication history

The pre-publication history for this paper can be accessed here:


